# Platelet Activation in High D-Dimer Plasma Plays a Role in Acquired Resistance to Epidermal Growth Factor Receptor Tyrosine Kinase Inhibitors in Patients with Mutant Lung Adenocarcinoma

**DOI:** 10.3389/fonc.2022.876051

**Published:** 2022-06-08

**Authors:** Meng-Jung Lee, Chih-Ming Weng, Wei Chao, Yueh-Fu Fang, Fu-Tsai Chung, Chien-Huang Lin, Han-Pin Kuo

**Affiliations:** ^1^ Graduate Institute of Medical Sciences, College of Medicine, Taipei Medical University, Taipei, Taiwan; ^2^ Thoracic Medicine Research Center, Taipei Medical University, Taipei, Taiwan; ^3^ School of Respiratory Therapy, College of Medicine, Taipei Medical University, Taipei, Taiwan; ^4^ Department of Thoracic Medicine, Chang Gung Medical Foundation, Chang Gung University College of Medicine, Taipei, Taiwan; ^5^ Department of Thoracic Medicine, Taipei Medical University Hospital, Taipei, Taiwan

**Keywords:** epidermal growth factor receptor, tyrosine kinase inhibitor, lung adenocarcinoma, platelet, epithelial–mesenchymal transition, D-dimer, Src, Akt

## Abstract

**Objective:**

Platelet activation and adhesion to cancer cells increase the release of multiple factors that contribute to EMT and chemoresistance. Elevated levels of D-dimer have been associated with poor clinical outcomes in lung cancer. Platelets in high D-dimer plasma may be activated and implicated in acquired resistance to EGFR TKI in advanced lung adenocarcinoma with mutant EGFR.

**Materials and Methods:**

Clinical responsive rate (RR), progression-free survival (PFS), and overall survival (OS) were prospectively measured in treatment-naïve lung adenocarcinoma patients with activation mutation. Plasma or platelets from patients with high or low D-dimer level were obtained to investigate the cytotoxic effects of TKIs on mutant cancer cells, and the mechanistic pathways were also explored.

**Results:**

Patients with high D-dimer had worse RR, PFS, and OS. High D-dimer plasma induced resistance to gefitinib, erlotinib, afatinib, or osimertinib in EGFR mutant lung cancer cells. Depletion of platelets in high D-dimer plasma reversed the resistance to TKI. Platelets of high D-dimer plasma had higher adherence capacity to cancer cells, and induced EGFR and Akt activation as well as EMT through Src activation. Inhibition of platelet adherence or activation of Src or Akt conquered the resistance to TKI. The acquired resistance to TKI by high D-dimer plasma was less attributed to secondary gene mutation.

**Conclusion:**

Increased platelet activation in the high D-dimer plasma may contribute to first-line acquired EGFR TKI resistance. Thus, therapeutic strategy against platelet activation in patients with high D-dimer levels may improve the efficacy of first-line treatment with EGFR TKI.

## Introduction

Blockage of dysregulated EGFR with tyrosine kinase inhibitors (EGFR-TKI) has played a central role in the treatment of advanced non-small cell lung cancer (NSCLC) with a significant improvement in clinical outcome: a response rate as high as 80%, especially for lung cancer patients with exon 19 deletions or an L858R mutation. However, acquired resistance and secondary progression are seen in almost all the patients with a median of 10–14 months of progression-free survival (PFS) (1–3). Molecular mechanism analysis reveals that the T790M point mutation, which lowers TKI binding affinity to the ATP pocket, is the most frequent underlying mechanism (4, 5), though it is more frequent with reversible TKI (gefitinib and erlotinib) than irreversible afatinib (6). Less frequent resistance mechanisms include ERBB2 and MET amplifications (7, 8) and mutations within the downstream signaling molecules BRAF, KRAS, PIK3CA, and CTNNB1 (9). Nevertheless, the efficacy differs a lot among patients with the same EGFR-sensitive mutations (10). The histological transformation into small cell or sarcomatoid lung cancer phenotypes, aberrations of drug transporters, or lysosomal sequestration (11) has been reported for the mechanisms underlying the diminished efficacy of EGFR TKI. However, given the multiple possible escape strategies, it remains a big challenge to predict the future mechanism of resistance of a specific tumor and target it from the beginning.

Increasing evidence has demonstrated that platelets play an important role in cancer survival, growth, and metastasis (12, 13). Within the blood circulation, tumor cells can aggregate with platelets and avoid cytotoxicity of natural killer cells (14, 15), indicating that platelet adhesion to tumor cells is a crucial step for tumor cell survival within the blood circulation. Direct contact of platelets with tumor cells also results in activation (16). Platelet–tumor cell aggregates form through binding of platelet integrin αIIbβ3 to tumor cell integrin αvβ3 *via* RGD-containing proteins including fibrinogen, von Willebrand factor, and fibronectin, a process known as tumor cell-induced platelet aggregation (TCIPA) (10). Once activated, platelets release an array of biologically active molecules that can modulate tumor growth, angiogenesis, and metastasis, including transforming growth factor beta (TGF-β1), vascular endothelial growth factor (VEGF), and platelet-derived growth factor (PDGF), inducing epithelial mesenchymal transit (12, 13). The roles of platelets in tumor development have also been shown to contribute to chemoresistance (17, 18).

D-dimer is a dimerized fragment from fibrinogen and a marker of thrombin activity and fibrin turnover, and represents both hemostasis and fibrinolysis (19). A variety of cancers have association between D-dimer and clinical manifestations such as tumor stage, metastasis and growth, and progression of cancers, as well as thromboembolic events (20, 21). D-dimer levels are a useful predictor for survival independent of clinical stage, histologic tumor type, and performance status of lung cancer patients (22). The mechanism underlying the relationship between D-dimer levels and lung cancer prognosis remains unknown. It is well known that platelet activation and blood coagulation are complementary, mutually dependent processes in hemostasis and thrombosis (23). Platelets interact with several coagulation factors, while the coagulation product thrombin is a potent platelet-activating agonist (23). Thus, the platelets in patients with high D-dimer may be further activated. On the other hand, the enhanced fibrin formation and fibrinolysis in cancer patients with high D-dimer may be secondary to platelet activation and aggregation (24).

This study addressed the question whether platelets in high D-dimer plasma of patients with mutant adenocarcinoma conferred EGFR-TKI acquired resistance. The results of this prospective study revealed that platelets were more activated in patients with high plasma D-dimer levels contributing to the development of phosphorylation of EGFR and Akt, as well as epithelial–mesenchymal transition (EMT) through Src activation, resulting in poor PFS and overall survival (OS). The acquired resistance to EGFR TKI in high D-dimer plasma was less attributed to secondary gene mutation. Thus, therapeutic strategy against platelet activation in patients with high D-dimer levels may improve the efficacy of first-line treatment with EGFR TKI.

## Materials and Methods

### Subjects’ Characteristics

During 2016 to 2018, 102 late-stage (Stage IV) treatment-naïve, non-smoking patients with mutant adenocarcinoma (Exon19 deletion or exon 21 point-mutation) without primary T790M or *ERBB2* and *MET* amplifications or ALK and ROS1 rearrangement intending to receive EGFR TKI treatment (gefitinib, erlotinib, or afatinib) were recruited from the outpatient department of Chang Gung Memorial Hospital and Taipei Medical University Hospital (both were tertiary referral hospitals in Northern Taiwan) into this 2-year prospective observational study. The biopsied specimens of naïve lung cancer that were routinely screened for mutation analysis of EGFR (exon18-21) were analyzed, including exon 19 deletions and L858R and T790M missense mutations by PCR assays with the Cobas EGFR mutation test. ERBB2 amplification and MET fusion or variant transcript were detected by RNA sequencing, and ALK and ROS1 rearrangement was confirmed by immunohistochemistry (IHC) assay with anti-ALK and anti-ROS1 rabbit monoclonal primary antibodies (VENTANA). The levels of D-dimer were measured before treatment. Patients with evident deep vein thrombosis, under anti-coagulant and/or anti-platelet treatment, with symptomatic heart failure (>NYHA II), with prior or coexistence of other malignancies, or with GOLD stage III-IV COPD were excluded from the recruitment. The existence of deep vein thrombosis in patients with high D-dimer levels was systemically assessed, using duplex ultrasonography and CT angiography. Clinical responses were assessed by response rate, PFS, and OS. Re-biopsy of tumors after disease progression was performed in most patients. Genetic analysis of the mechanisms for secondary resistance to EGFR TKI was also done. Because osimertinib was not reimbursed by the National Health Insurance in Taiwan during this study period, most patients received platinum-based doublet chemotherapy after disease progression, while 10 patients in the low D-dimer group and 6 patients in the high D-dimer group received self-pay osimertinib treatment for disease progression with the 2nd T790M mutation. Since osimertinib has been shown to be effective in counteracting with resistance T790M, those patients with osimertinib treatment were excluded from the OS assessment. All patients provided informed consent to participate in this study, which was approved by the local ethics committee [IRB was provided by the TMU-Joint Institutional Review Board (no. N201808072)].

### Proteomic Analysis of High D-Dimer Plasma

The proteomic analysis of patient’s plasma was performed by Biotools service (Biotools Co., Ltd., Taiwan) according to the manufacturer’s instructions.

### Preparation of Platelet-Rich Plasma and Platelet-Poor Plasma

Plasma was separated from the whole blood of high or low D-dimer patients. In brief, after centrifugation, the yellow upper phase containing the plasma component was transferred to new tubes with great care and then centrifuged again. The lower one-third was the platelet-rich plasma (PRP) and the upper two-thirds was the platelet-poor plasma (PPP). At the bottom of the tube, platelet pellets were formed (25).

### Cell Lines and Cell Viability Assay

HCC827 (Cat# CRL-2868) and NCI-H1975 (Cat# CRL-5908) were purchased from the American Type Culture Collection (ATCC), and PC9 (Cat# 90071810) was purchased from Sigma-Aldrich Corporation (St. Louis, MO). The cancer cells were cultured in high-glucose RPMI with 10% FBS and antibiotics in a humidified 37°C incubator, and seeded onto 96 wells for cell viability assay and onto 6-cm dishes for transfection and immunoblotting.

### Surface Protein Analysis of Platelet

PRP was isolated from normal, low, or high D-dimer lung cancer patients and then stained with CD42b-PE and glycoprotein VI-AF647 for 30 min. After washing, the percentage of CD42b^+^/GPVI^+^ cells was analyzed by a FACSLyrics flow cytometer and FACSuite software (Becton Dickinson, Mountain View, CA, USA).

### Platelet Adherence to Cancer Cells

The PRP of patients with low and high D-dimer levels was added to cultured HCC827 cells for 2 h in the presence or absence of PGI2 or dasatinib. Platelets were labeled with CD42b-PE, and non-adherent platelets were washed with PBS. The adherent platelets were counted under high-power fields of fluorescence microscopy for a total of 5 fields.

### Transfection of E-Cadherin siRNA

E-cadherin (GenBank no. NM_004360) siRNAs were generated, following the sequence of siRNA1: 5’-GGGUUAAGCACAACAGCAA-3’ and siRNA2: 5’-CAGACAAAGACCAGGACUA-3’. HCC827 cells were transfected with siRNA against E-cadherin using the DharmaFect 1 transfection reagent for 6 h, and cells were then measured with MTT assay or Western blot analysis.

### Western Blot Analysis

Western blot analyses were performed as described previously (26). Briefly, whole-cell lysates (50 μg) were subjected to SDS-PAGE and transferred onto a polyvinylidene difluoride (PVDF) membrane. Proteins were visualized by specific antibodies and the immunoreactivity was detected using enhanced chemiluminescence (ECL) following the manufacturer’s instructions. Quantitative data were obtained using a computing densitometer with scientific imaging systems (Kodak, Rochester, NY).

### Statistical Analysis

The receiver operating characteristic (ROC) curve was used to estimate the D-dimer levels in predicting disease progression with EGFR TKI treatment. The Kaplan–Meier method was used to estimate the distribution of survival curves, and log-rank tests were used to compare the distributions between groups. One-way analysis of variance (ANOVA) followed by Dunnett’s test, where appropriate, was used to determine the statistical significance of the difference between means for the results of *in vitro* cell line studies. Values of *p* less than 0.05 were considered statistically significant.

## Results

The ROC curve for the D-dimer assay in the prediction of disease progression is shown in **Supplementary Figure S1**. The cutoff values for the D-dimer levels were determined to be 0.82 μg/ml based on the ROC curve. The area under the curve was 0.8063 ± 0.0472, *p* < 0.0001 (*N* = 40). Based on the cutoff values from the ROC curve, patients were divided into two groups, high and low D-dimer groups.

### Clinical Characteristics

**Figure 1:**
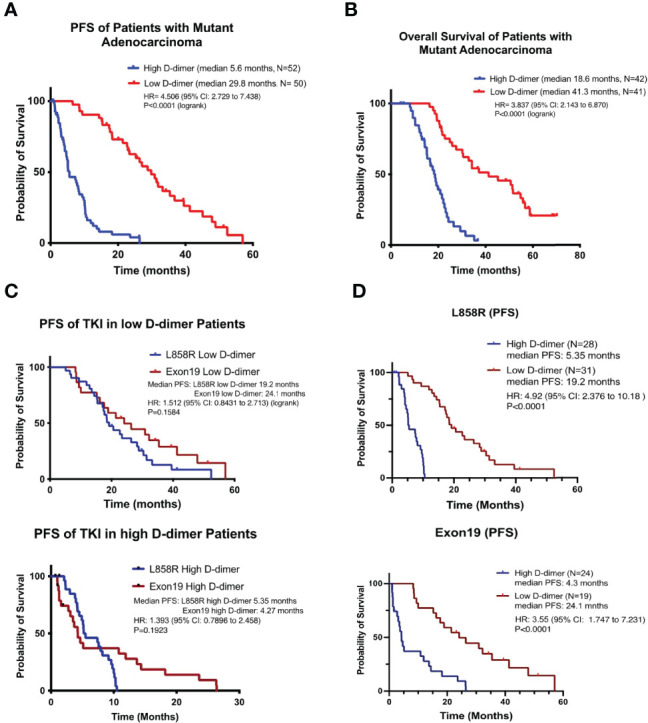
NSCLC patients with high D-dimer have poor free survival rate. The Kaplan–Meier survival curves of progression-free survival **(A)** and overall survival **(B)** or L858R **(C)** or exon19 del **(D)** genotypes of patients with high or low level of D-dimer in target therapy. *p*-value was compared to the low D-dimer group.

There was no significant difference in clinical characteristics between the high (*N* = 52) and the low (*N* = 50) D-dimer groups of patients, but there was a significantly higher level of fibrinogen in the high D-dimer group (**Table 1**). Patients in the high D-dimer group had a lower clinical response rate (34.6%, *N* = 52, *p* < 0.02) and a worse PFS (median 5.6 months, *p* < 0.0001, *N* = 52) to TKI treatment compared to the low D-dimer group (76%, *N* = 50; median 29.8 months, respectively, *N* = 50) (**Table 1**, **Figure 1A**) with a hazard ratio (HR) of 4.506 (95% CI: 2.729 to 7.438, Log-rank). The disease control rate was also favored in the low D-dimer group of patients (100% vs. 25%). Cox proportional HR analysis of clinical variables showed that high and low D-dimer levels and clinical response rate were independent variables for patients’ PFS (**Supplementary Table 1A**). Patients in the high D-dimer group had a worse OS (median 18.6 months, *N* = 42) compared with those in the low D-dimer group (median 41.3 months, *N* = 41, *p* < 0.0001) with an HR of 3.837 (95% CI: 2.143 to 6.870) (**Figure 1B**). Cox proportional HR analysis of clinical variables showed that high and low D-dimer levels and performance status were independent variables for patients’ OS (**Supplement Table 1B**).

**Table 1:** Clinical Characteristics of lung cancer patients.

	High D-dimer (*N* = 52)	Low D-dimer (*N* = 50)	*p*-value
Age (years)	66.9 ± 2.0	60.5 ± 1.5	0.153^¶^
Gender (M/F)	18/34	15/35	0.675^§^
Mutation L858R Exon 19	28 24	31 19	NS^§^
TKI Iressa Tarceva Afatinib	8 18 26	2 23 25	0.123^§^
Performance status	0.42 ± 0.10	0.27 ± 0.12	0.329^¶^
Response PR+CR SD+PD	18 34	38 12	<0.001^§^
D-dimer level (ng/dl)	2477.0 ± 436.6	312.2 ± 28.2	0.0006^¶^
Fibrinogen (mg/dl)	429.8 ± 28.7	329.5 ± 28.2	0.019^¶^
Thrombin time (s)	17.37 ± 0.32	17.9 ± 0.40	0.312^¶^
Platelet count (count ×10^3^/ml)	245.8 ± 21.4	259.2 ± 14.6	0.66^¶^

Data are means ± SEM. ^§^Chi-square test. ^¶^Unpaired t-test.

There was no significant difference between patients with the exon19 or L858R genotype in the proportion of patients with high D-dimer (**Table 1**) or PFS of EGFR TKI treatment (**Figures 1C, D**). There was no significant difference in PFS between exon19 and L858R genotypes of patients in the high D-dimer groups (median 4.27 months, *N* = 28; vs. median 5.35 months, *N* = 24, *p* = 0.192) or in the low D-dimer groups (median 24.1 months, *N* = 31 vs. 19.2 months, *N* = 19, *p* = 0.158) (**Figures 1C, D**). Neither was there a significant difference in PFS or OS between 1st-generation TKI (Tarceva and Iressa) and 2nd-generation TKI (afatinib) treatment groups (**Supplementary Figure S2**).

Re-biopsy of tumor in patients with disease progression revealed that a higher proportion of patients in the low D-dimer group had the T790M mutation (61.9%, *N* = 21) compared to patients in the high D-dimer group (19.4%, *N* = 31, *p* = 0.018, Chi-square test). In contrast, EGFR mutation persisted their genotypes in patients in the high D-dimer group (80.6%, *N* = 31) compared to patients in the low D-dimer group (38.1%, *N* = 21) (**Supplementary Figure S3**). Among those with the 2nd T790M mutation, 10 from the low D-dimer group and 6 from the high D-dimer group received self-pay osimertinib treatment. One from the low D-dimer group lost to follow-up. Nine from the low D-dimer group and 3 from the high D-dimer group had clinical response to osimertinib, while 3 from the high D-dimer group failed to significantly respond to the treatment (*p* = 0.044, Fisher’s exact test, **Supplementary Figure S4**). Patients in the low D-dimer group had better survival benefit to osimertinib treatment in terms of PFS (median 21.0 months, *N* = 9, *p* = 0.0109) and OS (median 36.2 months, *N* = 8, *p* = 0.0288), compared with the high D-dimer group (median 7.0 months, *N* = 6 and 20.5 months, *N* = 6, respectively) (**Supplement S4**).

### Plasma From the High D-Dimer Patients Induced Resistance to TKI in MutantNon-Small Cell Lung Cancer Cells

**Figure 2:**
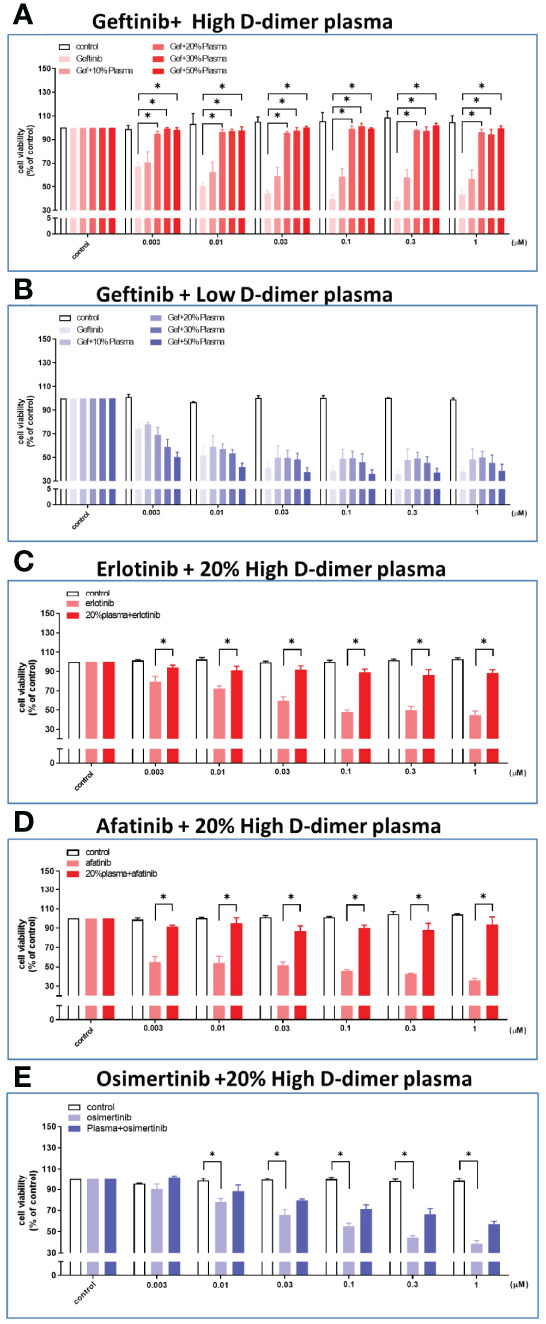
Plasma from high D-dimer NSCLC patients induced tyrosine kinase inhibitor resistance in HCC827 cells. The HCC827 cells in 96-well plates were treated with different concentrations of the patient’s plasma from the high D-dimer level **(A)** and the low D-dimer level **(B)** for 6 h, and then incubated with different concentrations of gefitinib for 72 (h) After incubation, the MTT was added in culture medium for 2 h, and the absorbance was read at 570 nm. Data represent the mean ± SEM of five experiments, with the vehicle control as the 100% reference. The HCC827 cells in 96-well plates were preincubated with 20% high D-dimer plasma for 6 h, and then treated with different concentrations of erlotinib **(C)** or afatinib **(D)** or osimertinib **(E)** for 72 h, and MTT was added in culture medium for another 2 (h) The absorbance was read at 570 nm. Data represent the mean ± SEM of three experiments, with the vehicle control as the 100% reference. *p < 0.05 compared to corresponding vehicle control or gefitinib group.

Plasma collected from patients in either the high or the low D-dimer group was diluted as indicated with culture medium before incubation with HCC827 cells. The plasma from patients of the high D-dimer group induced more than 90% resistance to gefitinib treatment (up to 1 μM) after incubation for 72 h at the concentrations ≥20% (**Figure 2A**), but not those from the low D-dimer group (**Figure 2B**). The following studies adopted 20% plasma for experiments. The 20% plasma of the high D-dimer group also induced HCC827 cells >90% resistance to either erlotinib or afatinib treatment (**Figures 2C, D**). In contrast to the 1st- and 2nd-generation EGFR TKI, the 3rd-generation TKI, osimertinib, could still induce cytotoxicity approximately 50% in the presence of high D-dimer plasma (**Figure 2E**). High D-dimer plasma also induced resistance to gefitinib in PC9 and H1975 cells (**Supplementary Figure S5**).

### Platelets in High D-Dimer Plasma Induced Resistance to EGFR TKI

**Figure 3:**
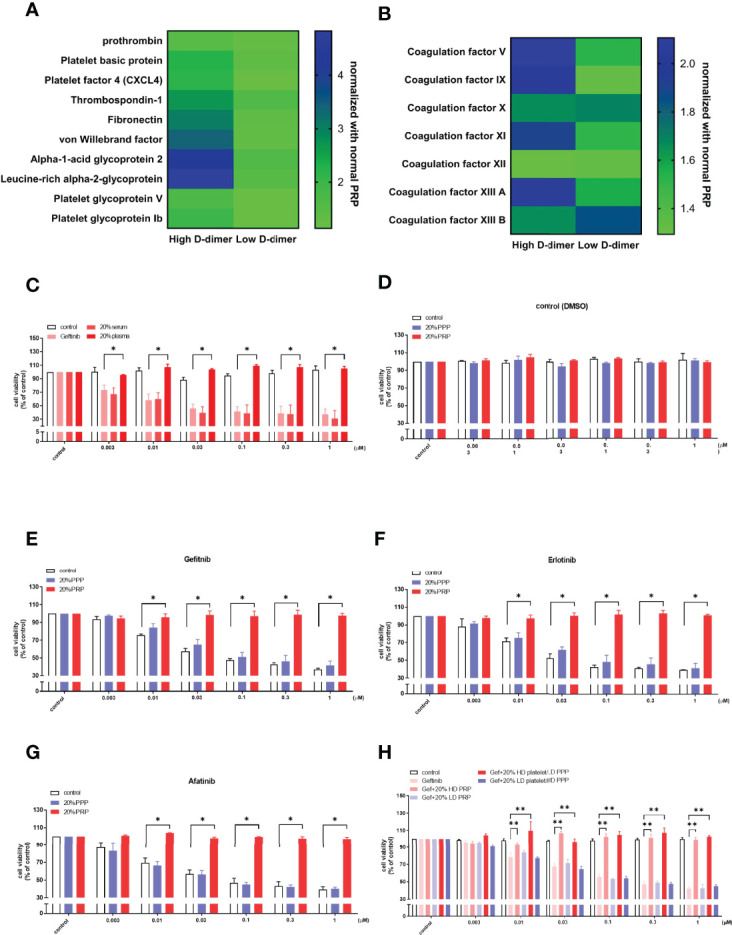
Platelets play a crucial role in plasma-induced TKI resistance in HCC827. Heatmap showing the proteomic results of platelet-rich plasma from high D-dimer or low D-dimer NSCLC patients was analyzed for platelet activation factors and coagulation factors **(A, B)**. Columns indicate biological replicates from the experiments (blue, high; yellow, low). **(C)** The HCC827 cells in 96-well plates were treated with 20% of the patient’s plasma or serum from the high D-dimer level for 6 h, and then incubated with different concentrations of gefitinib for 72 h, the MTT was added in the culture medium for 2 h, and the absorbance was read at 570 nm. The HCC827 cells in 96-well plates were preincubated with 20% high D-dimer platelet-rich plasma (PRP) or platelet-poor plasma (PPP) for 6 h, and then treated with DMSO as the controls **(D)** or with different concentrations of gefitinib **(E)**, erlotinib **(F)**, or afatinib **(G)** (*N* = 3, respectively) for 72 h; the MTT was added in culture medium for 2 h, and the absorbance was read at 570 nm. **(H)** The HCC827 cells in 96-well plates were treated with 20% high or low D-dimer plasma, or high or low D-dimer platelets for 6 h, and then incubated with different concentrations of gefitinib (*N* = 3) for 72 h. The MTT was added in culture medium for 2 h, and the absorbance was read at 570 nm. Data represent the mean ± SEM of three experiments, with the vehicle control as the 100% reference. *p < 0.05; **p < 0.01 compared with the corresponding TKI treatment group.

Proteomic analysis of the pooled high and low D-dimer plasma revealed an increase in pro-coagulation factors (factors V, IX, and XI) that led to thrombin, fibrin clot formation, and cross-link (XIIIa); factors that promote platelet aggregation and adherence (fibronectin, von Willebrand factor, platelet glycoprotein Ib, thrombospondin-1, and leucine-rich alpha 2 glycoprotein); and factors released from activated platelets (platelet factor 4 and platelet basic protein) (**Figures 3A, B**). Sera of the high D-dimer group failed to induce resistance to gefitinib in HCC827 cells (**Figure 3C**). To determine the role of platelets in inducing TKI resistance, plasma of the high D-dimer group was prepared as PRP and PPP. PPP failed to induce resistance to gefitinib, erlotinib, or afatinib, compared with PRP (**Figures 3D–G**). To further exclude the influence of humoral factors on high D-dimer plasma induced TKI resistance, platelets of the high D-dimer plasma were replaced by concentrated platelets from the low D-dimer plasma (LD platelet/HD PPP), or platelets of the low D-dimer plasma were replaced by concentrated platelets from the high D-dimer plasma (HD platelet/LD PPP). **Figure 3H** reveals that HD platelet/LD PPP induced resistance to gefitinib to the same extent as the high D-dimer plasma. In contrast, LD platelet/HD PPP failed to induce any resistance.

**Figure 4:**
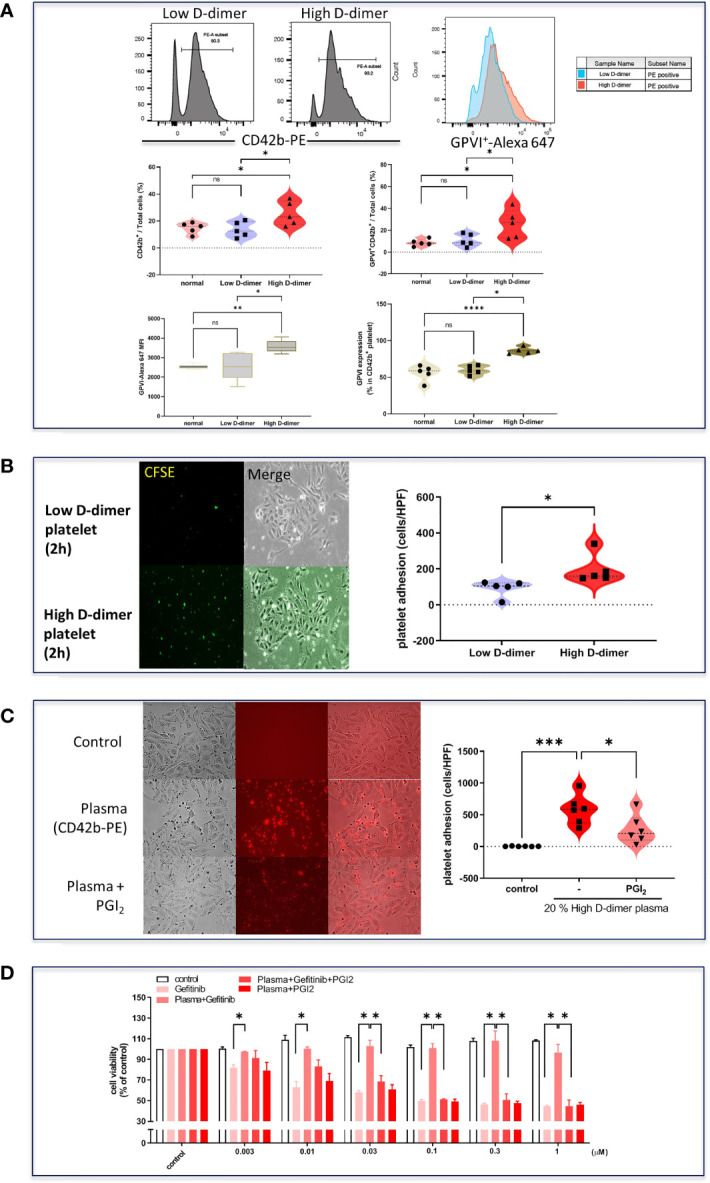
Increased cell adhesion and surface protein glycoprotein VI in high D-dimer platelets. **(A)** Isolated platelets from low D-dimer and high D-dimer patients were stained with specific antibodies for CD42b-PE and glycoprotein VI-AF647 and then analyzed by flow cytometry. The histogram of flow cytometry analysis for surface protein expression and MFI in platelets isolated from patients with low or high D-dimer. Data represent the mean ± SEM of five patients. *p < 0.05, **p < 0.01, ***p < 0.005, ****p < 0.001 compared to the corresponding vehicle control or low D-dimer group. ns, not significant. **(B)** Isolated platelets from low D-dimer and high D-dimer patients were labeled with CFSE-DA and then incubated with HCC827 for 2 h, followed by washing twice with PBS. The images of platelet adhesion were recorded by a fluorescence microscope, and the statistical results were calculated as the average of five HPF from five patients. Data represent the mean ± SEM of five patients. *p < 0.05 compared to the low D-dimer group. **(C)** Isolated platelets from high D-dimer patients were treated with or without prostacyclin (PGI_2_) for 30 min and then labeled with CD42b-PE. After labeling, isolated platelets were incubated with tumor cells for another 1 h, and washed three times with PBS. The images of platelet adhesion were recorded by a fluorescence microscope, and the statistical results were calculated as the average of five HPF from six patients. *p < 0.05, ***p < 0.005 compared to the corresponding vehicle control or high D-dimer group. **(D)** The isolated platelets were treated with PGI_2_ for 30 min and then incubated with HCC827 cells for 6 h in 96-well plates. After incubation, cells were treated with different concentrations of gefitinib for 72 h, and the MTT was added in culture medium for 2 h, and the absorbance was read at 570 nm. Data represent the mean ± SEM of three experiments. *p < 0.05 compared to the corresponding gefitinib or high D-dimer group.

Isolated platelets from the high D-dimer plasma were found to increase the expression of surface protein GPIb-V-XI (CD42b) (**Figure 4A**) and adherence to tumor cells, compared with platelets from the low D-dimer plasma (**Figure 4B**). The GPVI-Alexa 647 MFI and the proportion of GPVI^+^ of CD42b^+^ platelets were higher in the high D-dimer plasma, compared to the low D-dimer plasma (**Figure 4A**). Pretreatment with prostacyclin to inhibit platelet adherence (**Figure 4C**) almost completely reversed the high D-dimer plasma-induced resistance to gefitinib (**Figure 4D**).

### Signaling Pathways Underlying High D-Dimer Plasma-Induced EGFR-TKI Resistance

**Figure 5:**
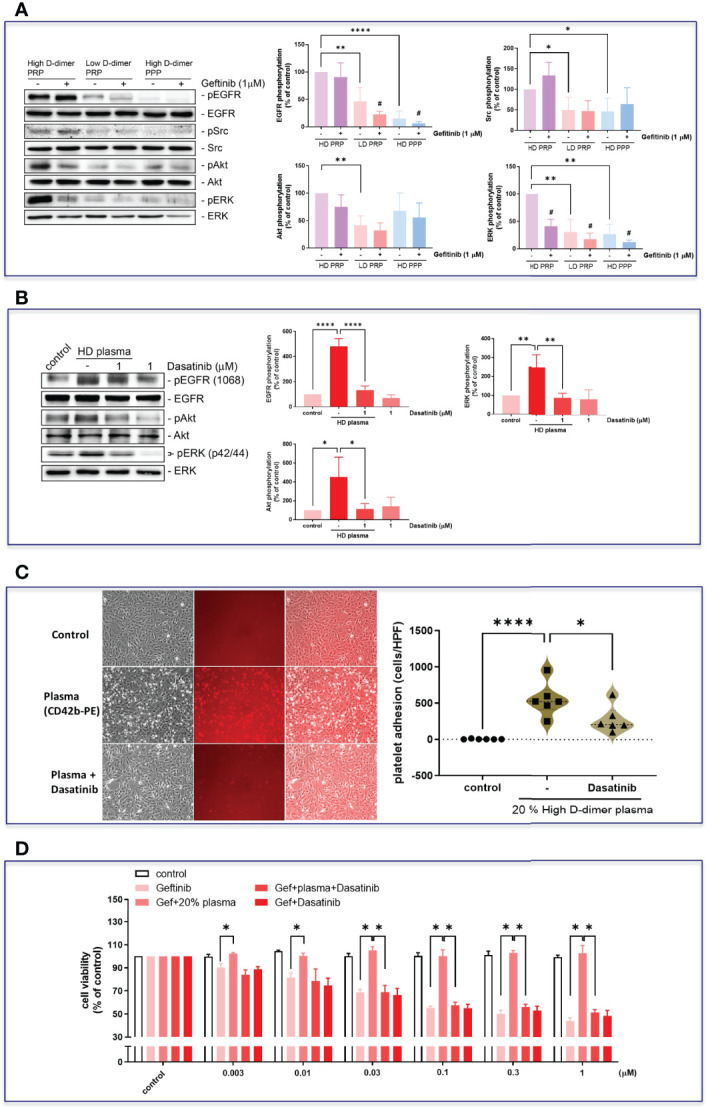
Plasma induced EGFR-related signal activation in HCC827 *via* Src. **(A)** The HCC827 cells in 6-cm dishes were treated with or without 1 μM gefitinib for 30 min and then incubated with 20% high D-dimer PRP, low D-dimer PRP, or high D-dimer PPP for 24 h; Western blot analysis was performed, and proteins were detected by specific antibodies for the phosphorylation form of EGFR, Src, Akt, or ERK. Data represent the mean ± SEM of four experiments.*p < 0.05, **p < 0.01, ****p < 0.001 compared to the HD PRP group as the 100% reference; ^#^ *p* < 0.05 compared with the corresponding control. The exposure time of the bands of Western blot was reduced to avoid overexposure of the bands of high D-dimer PRP, resulting in a reduced expression in low D-dimer PRP group and high D-dimer PPP. **(B)** HCC827 cells were pretreated with dasatinib (1 μM) and then cells were treated with 20% high D-dimer plasma for 24 h. Western blot analysis was performed, and proteins were detected by specific antibodies for the phosphorylation form of EGFR, Akt, or ERK. Data represent the mean ± SEM of three experiments. *p < 0.05, **p < 0.01, ****p < 0.001 compared to the corresponding vehicle control or HD plasma group. The exposure time of the bands of Western blot was reduced to avoid overexposure of the bands of high D-dimer plasma, resulting in a reduced expression in the control. **(C)** Isolated platelets from high D-dimer patients were treated with or without dasatinib for 30 min and then labeled with CD42b-PE. After labeling, isolated platelets were incubated with tumor cells for another 1 h, and washed three times with PBS. The images of platelet adhesion were recorded by a fluorescence microscope, and the statistical results were calculated as the average of five HPF from six patients. *p < 0.05, ****p < 0.001 compared to the corresponding vehicle control or HD plasma group. **(D)** The isolated platelets were treated with dasatinib for 30 min and then incubated with HCC827 cells for 6 h in 96-well plates. After incubation, cells were treated with different concentrations of gefitinib for 72 h, the MTT was added in culture medium for 2 h, and the absorbance was read at 570 nm. Data represent the mean ± SEM of three experiments. *p < 0.05 compared to the gefitinib or HD plasma group.

To examine the role of platelets in high D-dimer plasma in inducing EGFR-TKI resistance, platelet depletion in high D-dimer plasma (high D-dimer PPP) acted as the negative control for high D-dimer PRP, in which platelets were enriched. Low D-dimer PRP also examined the effects of enriched platelets in comparison with high D-dimer PRP. The PRP of the high D-dimer plasma, but not those from PRP of the low D-dimer plasma or PPP of the high D-dimer plasma, induced phosphorylation of EGFR and Src, and the downstream signal pathways, ERK and Akt (**Figure 5A**). In the presence of gefitinib, phosphorylation of EGFR was suppressed in PRP of the low D-dimer plasma or PPP of the high D-dimer plasma-treated HCC827 cells (**Figure 5A**). Gefitinib also almost completely inhibited ERK phosphorylation (**Figure 5A**). However, gefitinib failed to suppress EGFR or Src or Akt phosphorylations induced by the PRP of the high D-dimer plasma group (**Figure 5A**). An Src inhibitor (dasatinib) completely suppressed EGFR, Akt, and ERK phosphorylation induced by high D-dimer plasma (**Figure 5B**) and also significantly inhibited platelets of the high D-dimer plasma adherence to HCC827 cells (**Figure 5C**). PRP of the high D-dimer-induced TKI resistance was also reversed by dasatinib (**Figure 5D**).

**Figure 6:**
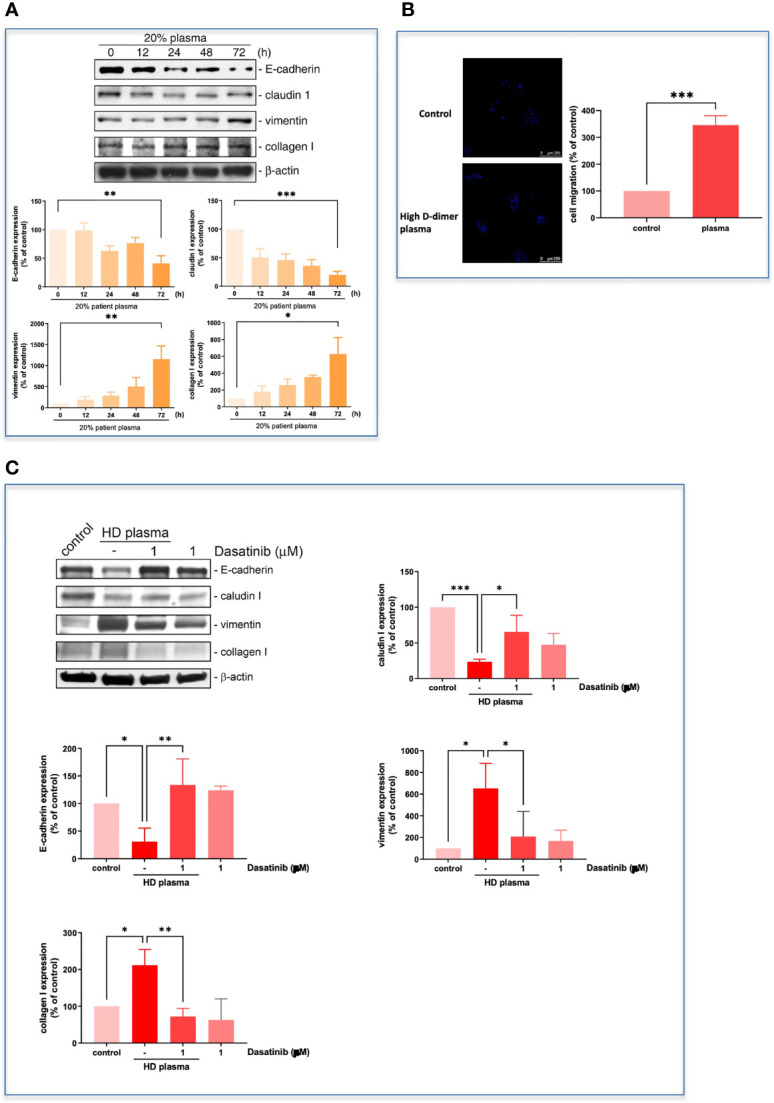
Plasma induced EMT progression in HCC827. **(A)** The HCC827 cells in 6-cm dishes were incubated with 20% high D-dimer plasma for different time intervals. Western blot analysis was performed, and proteins were detected by specific antibodies for EMT markers. The data were calculated and represent the mean ± SEM of three experiments shown in statistical figures. *p < 0.05, **p < 0.01, ***p < 0.005 compared to the corresponding vehicle control as the 100% reference. **(B)** HCC827 cells in 8 μM transwells were incubated with 20% high D-dimer plasma for 24 (h) The membrane of transwells was cut, stained with Hoechst33342, and then counted for positive cells under fluorescence microscopy. The data were calculated and represent the mean ± SEM of three experiments shown in statistical figures. ***p < 0.005 compared to the corresponding vehicle control as the 100% reference. **(C)** HCC827 cells were pretreated with dasatinib (1 μM) and then cells were treated with 20% high D-dimer plasma for 24 h. Western blot analysis was performed, and proteins were detected by specific antibodies for EMT markers. Data represent the mean ± SEM of three experiments. *p < 0.05, **p < 0.01, ***p < 0.005 compared to the corresponding vehicle control or HD plasma treatment group.

**Figure 7:**
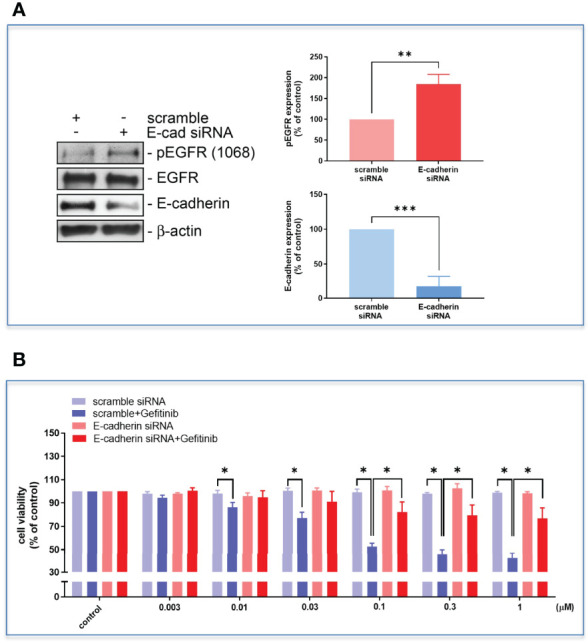
The HCC827 cells in 96-well plates were transfected with E-cadherin siRNA and then incubated with different concentrations of gefitinib, the Western blot **(A)** and MTT assay **(B)** were performed, and the absorbance was read at 570 nm. Data represent the mean ± SEM. *p < 0.05, **p < 0.01, ***p < 0.005 compared to the corresponding vehicle control or treated group.

The high D-dimer plasma also time-dependently induced a decrease in epithelial cell markers, and an increase in mesenchymal cell markers in HCC827 cells (**Figure 6A**). The EMT-transformed HCC827 cells increased their migratory activities compared to the controls (**Figures 6A, B**). Dasatinib significantly reversed the high D-dimer plasma-induced EMT (**Figure 6C**). To explore whether a loss of E-cadherin would induce EGFR activation, the E-cadherin siRNA was used to knock down E-cadherin. The E-cadherin defective cells showed upregulated EGFR phosphorylation (**Figure 7A**) and developed partial resistance to gefitinib (**Figure 7B**).

## Discussion

In the present study, we demonstrated that patients with mutant lung adenocarcinoma with high D-dimer levels in their peripheral blood were less responsive to EGFR TKI, were more vulnerable to develop early disease progression, and had shorter survival. Most of the secondary resistance in those patients was beyond secondary gene mutation. The platelets in high D-dimer plasma were activated and conferred resistance to EGFR TKI *via* Src activation to trans-activate EGFR and the Akt signal pathway. These results indicated that the D-dimer plasma levels could be a good predictor for early development of acquired resistance to EGFR TKI in the beginning of therapy. The platelets of the high D-dimer plasma may become a therapeutic target to improve the efficacy of EGFR TKI in patients with mutant lung adenocarcinoma.

Cancer cells through TCIPA can confer an advantage to the survival and growth of cancer cells, metastatic potential, evading the body’s immune system and shielding it from high shearing force (15, 27, 28). Proteomic analysis of the high and low D-dimer plasma revealed that the high D-dimer plasma contained increased levels of proteins that promote platelet aggregation and adherence, factors released from activated platelets, and increased levels of coagulation factors that led to thrombin, fibrin clot formation, and cross-link. Thus, the high D-dimer plasma provided a good environment for platelet aggregation and adherence. The expression of platelet GPIb-IX-V, GPIIb/IIIa, and P-selectin on the tight inter-junction between platelet and cancer cells is crucial for TCIPA (29, 30). In this study, platelets of the high D-dimer plasma were found with the upregulated expression of GPIb-IX-V and GPIIb/IIIa, indicating that those platelets were activated by TCIPA and ready for aggregation and adherence. Another surface glycoprotein expression of activated platelets, GPVI, a surface receptor belonging to the immunoglobulin superfamily, which principally binds collagen (31), was also upregulated on platelets of the high D-dimer plasma. Prostacyclin is the most potent known inhibitor of platelet aggregation (32) and has been shown to inhibit TCIPA (33). In this study, treatment with prostacyclin inhibited platelet adherence to tumor cells, and completely reversed platelets of the high D-dimer plasma-induced TKI resistance. These results suggested that platelet activation and adherence to tumor cells contributed to EGFR TKI resistance. The surface receptors of platelets may be a potential therapeutic target to conquer the resistance development in EGFR mutant lung cancer patients.

When platelets aggregate around cancer cells, clustering of these surface receptors in activated platelets may activate Src family kinases (SFKs) to release a variety of cytokines and growth factors, which have been implicated in cancer growth, progression, and escape from apoptosis when challenged with chemotherapy (18, 34, 35). In the present study, depletion of platelets from the high D-dimer plasma failed to cause EGFR TKI resistance, suggesting that the humoral factors in the high D-dimer plasma was not directly contributory to induce TKI resistance. In contrast, enriched platelets of the high D-dimer plasma (PRP) induced gefitinib-resistant phosphorylation of Src, EGFR, and Akt signaling pathways in HCC827 cells. Src binds to EGFR, resulting in a variety of downstream effects and an induction of survival and migration signaling pathways (36). This downstream pathway activation may provide a synergism with EGFR (37) for tumor cells to escape from EGFR TKI inhibition (11). The present study demonstrated that treatment with dasatinib inhibited platelet adherence to tumor cells, phosphorylation of EGFR and Akt, as well as ERK, resulting in complete reversal of the high D-dimer plasma-induced TKI resistance. These results suggest that Src activation through platelet interaction with HCC827 cells plays a central role in platelets of high D-dimer plasma-induced TKI resistance.

The PI3K/Akt signaling pathway plays an important role in regulating cell proliferation and maintaining the biological characteristics of malignant cells (38), and also mediates EMT (39). Although Akt activation *via* PI3K and ERK *via* Ras are the two principal downstream signaling pathways mediating the oncogenic effects of EGFR (40), the high D-dimer plasma-induced Akt phosphorylation was not inhibited by EGFR TKI or gefitinib, but by the Src inhibitor, dasatinib, suggesting that Akt phosphorylation was mostly beyond EGFR activation, but resulted from a direct involvement with SFKs (41). Akt activation has been shown to be a convergent, resistance-driving signaling event across a spectrum of EGFR-mutant NSCLCs with acquired resistance to EGFR TKIs caused by diverse underlying mechanisms, such as amplification, overexpression, and activation of MET, FGFR, EphA2, Mer, and AXL or the T790M mutation (42). Akt phosphorylation has also been shown to increase in the majority of EGFR-mutant patients prior to EGFR-TKI treatment and correlates with poor initial therapeutic responses (42). In the present study, Akt inhibitors significantly reversed the high D-dimer plasma-induced TKI resistance, indicating that Akt activation played an important role in SFK-mediated acquired EGFR inhibitor resistance.

The activation of SFK has also been shown to induce E-cadherin deregulation and associated EMT, which acquired resistance to TKIs (43–45). In NSCLC, clinical cancer specimens with acquired gefitinib resistance showed a decrease in E-cadherin and an increase in Hakai expression (46). The dual HDAC and HMGR inhibitor reverses E-cadherin expression, attenuates vimentin and stemness, and restores gefitinib sensitivity through an inhibition of the Src/Hakai and Hakai/E-cadherin interaction (46). Here, we showed that the high D-dimer plasma induced EMT in HCC827 cells by decreasing the expression of E-cadherin and claudin 1, and increasing the expression of vimentin and collagen 1. The high D-dimer plasma also increased HCC827 cell migratory activity. Dasatinib was also shown to inhibit the high D-dimer-induced EMT and HCC827 cell migration. Disruption of E-cadherin alone may result in reduced suppression of EGFR-dependent signaling pathways (47, 48), since E-cadherin has been shown to suppress intracellular signaling pathways, which regulate cell activation, proliferation, and differentiation (49). Our results also showed that E-cadherin knockdown by siRNA transactivated EGFR and became resistant to gefitinib treatment. E-cadherin loss may further exacerbate Src-induced aberrant EGFR activation. Thus, the reversal effect on EMT may cast an important role in the efficiency of dasatinib in restoring EGFR TKI responsiveness.

Disruption of the SFK pathway may therefore provide a method to overcome EGFR TKI resistance. However, several clinical trials with dasatinib in combination with EGFR TKI failed to overcome acquired TKI resistance (50, 51). The lack of clinical benefits of combined therapies is attributed to an incomplete abrogation of c-Src hyper-activation and the enrolment of molecular uncharacterized patients (51). The heterogeneity of lung cancer cells in expressing Src kinase activity and dependence of Src activation in regulation of cell growth may be differentially responsive to Src inhibition and also differentially vulnerable to Src activation and development of EGFR TKI resistance. Src activation by platelet adherence to tumor cells in patients with mutant adenocarcinoma may be a predictive biomarker of responses to Src inhibitors in conquering acquired TKI resistance.

Based on our cell line *in vitro* studies, osimertinib could induce cytotoxicity approximately 50% in the presence of high D-dimer plasma (**Figure 2E**). We propose that osimertinib as a first-line treatment for patients in the high D-dimer group may have a significant survival benefit compared with comparator EGFR TKI. However, whether the 50% resistance to osimertinib in *in vitro* studies could be clinically translated into a significant difference in survival benefit deserves further studies if the superior efficacy of osimertinib in lung adenocarcinoma with activating EGFR mutation (52, 53) would conquer the induction of resistance by high D-dimer plasma. As shown in the second-line treatment for resistance T790M in our limited number of patients, the efficacy of osimertinib was hindered by high D-dimer plasma in terms of response rate, PFS, and OS.

In conclusion, our study demonstrated that platelets in the high D-dimer plasma were activated and induced EGFR TKI resistance through Src-mediated EGFR transactivation, Akt activation, and EMT in patients with mutant lung adenocarcinoma. Platelet activation in high D-dimer plasma might play a role in acquired resistance to TKI and poor clinical outcomes. Inhibiting platelet or/and Src activation may be a potential therapeutic direction to improve the efficacy of EGFR TKI in patients with high D-dimer plasma levels.

## Data Availability Statement

The original contributions presented in the study are included in the article/**Supplementary Material**. Further inquiries can be directed to the corresponding author.

## Ethics Statement

The studies involving human participants were reviewed and approved by the TMU-Joint Institutional Review Board. The patients/participants provided their written informed consent to participate in this study.

## Author Contributions

Conception and design: M-JL, C-MW, and H-PK. Supervising the research: C-HL and H-PK. Performing the experiments including quality control: M-JL, C-MW, and WC. Clinical resource: Y-FF, F-TC, and H-PK. Analysis and interpretation: all authors. Drafting the manuscript: M-JL, C-MW, C-HL, and H-PK. All authors contributed to the article and approved the submitted version.

## Funding

This work is supported by Taiwan Ministry of Science and Technology grants 110-2314-B-038-150 and 110-2314-B-038-145.

## Conflict of Interest

The authors declare that the research was conducted in the absence of any commercial or financial relationships that could be construed as a potential conflict of interest.

## Publisher’s Note

All claims expressed in this article are solely those of the authors and do not necessarily represent those of their affiliated organizations, or those of the publisher, the editors and the reviewers. Any product that may be evaluated in this article, or claim that may be made by its manufacturer, is not guaranteed or endorsed by the publisher.
