# Supplementary material for: Platelet Activation in High D-Dimer Plasma Plays a Role in Acquired Resistance to Epidermal Growth Factor Receptor Tyrosine Kinase Inhibitors in Patients with Mutant Lung Adenocarcinoma
Source: Front Oncol. 2022 Jun 8;12:876051. doi: 10.3389/fonc.2022.876051 (PMC9214222; doi:10.3389/fonc.2022.876051)
Supplement: Supplementary file 1 [file DataSheet_1.docx]

Supplementary Material

**Table S1**

1. **Cox proportional hazards analysis (Variables for PFS)**

| **Variable** | **Cox Hazard ratio** | **95% CI (profile likelihood)** | **P value** |
| --- | --- | --- | --- |
| **D-dimer** | **16.84** | **4.680 to 82.92** | **<0.0001** |
| **Age in years** | **0.9908** | **0.9480 to 1.035** | **0.6756** |
| **mutation** | **0.5631** | **0.2378 to 1.299** | **0.1807** |
| **TKI** | **0.7943** | **0.3597 to 1.761** | **0.5674** |
| **performance** | **1.680** | **0.6869 to 3.881** | **0.2354** |
| **Response** | **61.80** | **10.71 to 1230** | **0.0002** |
| **platelet counts** | **0.9944** | **0.9883 to 1.000** | **0.0633** |

1. **Cox proportional hazards analysis (Variables for OS)**

| **Variable** | **Cox Hazard ratio** | **95% CI (profile likelihood)** | **P value** |
| --- | --- | --- | --- |
| **D-dimer** | **19.49** | **7.463 to 54.71** | **<0.0001** |
| **Age in years** | **1.014** | **0.9894 to 1.041** | **0.2647** |
| **mutation** | **1.427** | **0.7485 to 2.728** | **0.2791** |
| **TKI** | **1.411** | **0.8576 to 2.348** | **0.1779** |
| **performance** | **2.801** | **1.317 to 6.029** | **0.0075** |
| **Response** | **1.453** | **0.7497 to 2.889** | **0.2748** |
| **platelet counts** | **0.9987** | **0.9935 to 1.004** | **0.6070** |

**Figure S1. ROC curve for the D-dimer assay in the prediction of disease progression.** The cut-off values for the D-dimer levels was determined to be 0.82 μg/ml based on the ROC curve, and the sensitivity and specificity for disease progression prediction were 40.0% and 90.0%, respectively. The area under curve was 0.8063+0.0472, P<0.0001 (N=40).


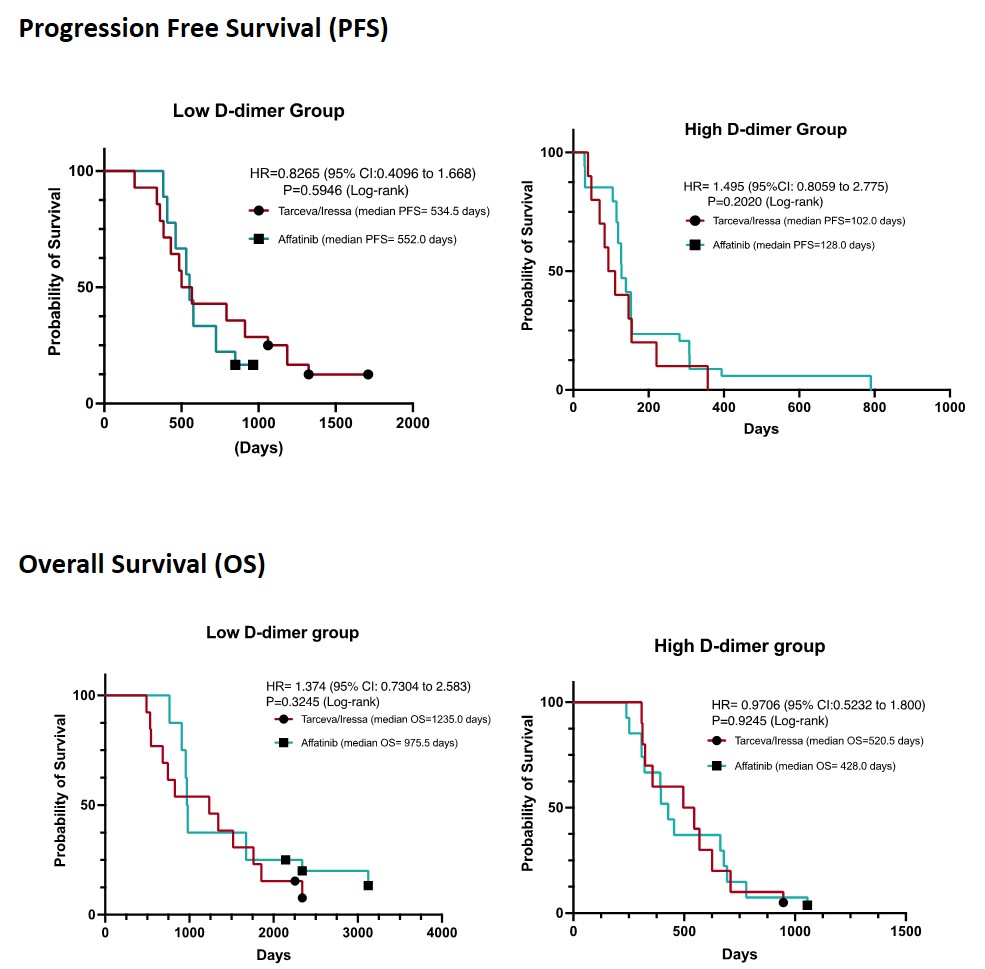
**Figure S2.** **The Kaplan–Meier survival curves of** **progression free survival** (**PFS) and overall survival (OS) in low or high D-dimer groups of mutant lung adenocarcinoma patients receiving 1st generation TKI (Tarceva and Iressa) and 2nd generation TKI (Affatinib) treatment.** P values were indicated, compared to corresponding affatinib treatment group.

**Figure S3 The proportion of genotype after progression** The genotype of EGFR mutant in adenocarcinoma patients with low or high D-dimer level were determined through real-PCR identification (N=52). The proportion of EGFR T790M mutation in low D-dimer group was 61.9% (N=21), and 19.4% in high D-dimer group (N=31). The EGFR mutation in patients in high D-dimer group was 77.4% (N=31) and 19.0% in low D-dimer group (N=21).


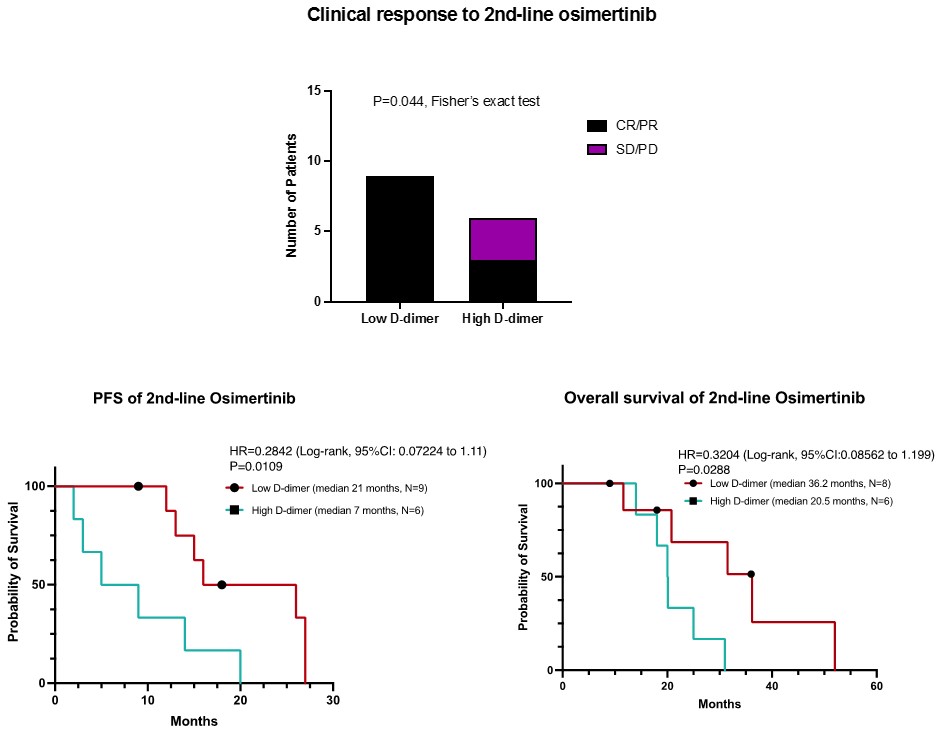
**Figure S4.** **Clinical responses and survival benefits to 2^nd^-line osimertinib in patients with resistance T790M.** Nine of low D-dimer group and 3 of high D-dimer group had clinical response to osimertinib in terms of complete regression (CR) or partial regression (PR), while 3 of high D-dimer group failed to significantly respond to the treatment in terms of stable disease (SD) or progressive disease (PD). Patients of low D-dimer group had better survival benefit to osimertinib treatment in terms of PFS and OS, compared with high D-dimer group. P values were indicated in the figures.


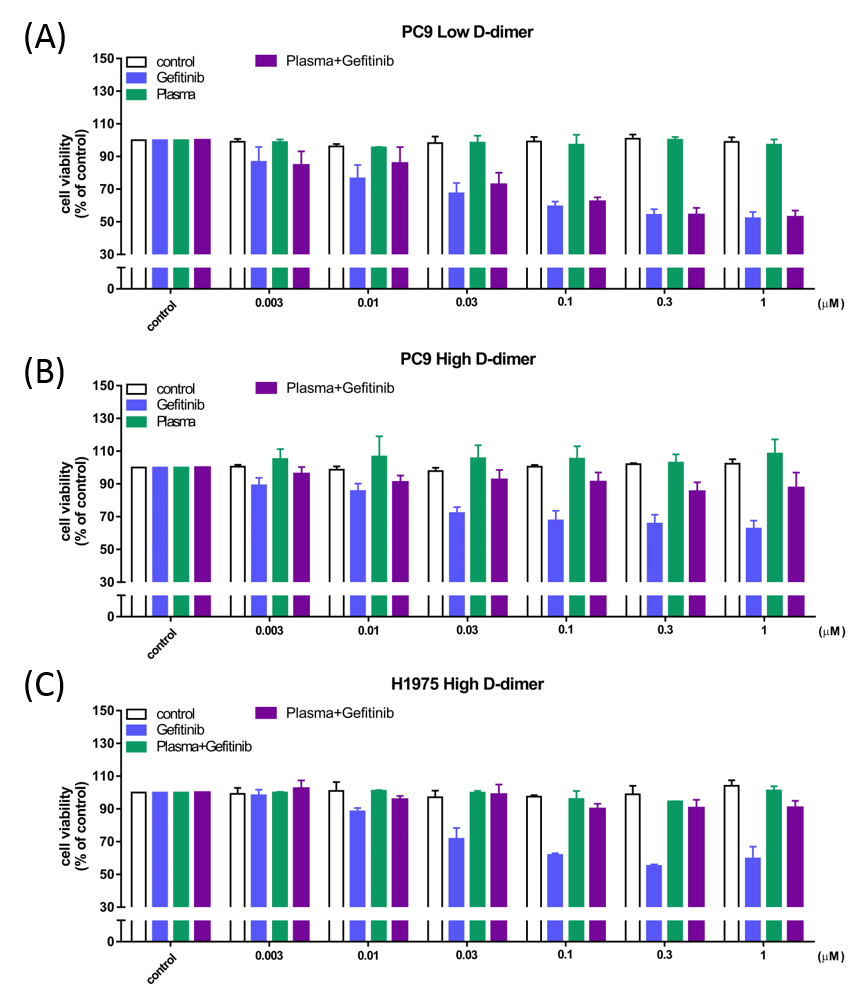


**Figure S5.** **Plasma of high D-dimer NSCLC patients also induced gefitinib resistance in EGFR-mutant lung cancer cell lines other than HCC827.** The PC9 or H1975 cells in 96 wells plate were treated with patient’s plasma from high D-dimer level or low D-dimer level for 6 h, and then incubated with different concentration of gefitinib for 72 h. After incubation, the MTT was added in culture medium for 2 h, and the absorbance was read at 570 nm. Data represent the mean ± SEM of three experiments, the vehicle control as the 100% reference.
